# 3D mobile regression vision transformer for collateral imaging in acute ischemic stroke

**DOI:** 10.1007/s11548-024-03229-5

**Published:** 2024-07-13

**Authors:** Sumin Jung, Hyun Yang, Hyun Jeong Kim, Hong Gee Roh, Jin Tae Kwak

**Affiliations:** 1 School of Electrical and Electronic Engineering, Korea University, Seoul, Republic of Korea; 2 Department of Radiology, St. Mary’s Hospital, College of Medicine, The Catholic University of Korea, Daejeon, Republic of Korea; 3 Department of Radiology, Konkuk University Medical Center, Konkuk University School of Medicine, Seoul, Republic of Korea

**Keywords:** Acute ischemic stroke, MRI, Collateral imaging, Lightweight model, Transformer

## Abstract

**Purpose:**

The accurate and timely assessment of the collateral perfusion status is crucial in the diagnosis and treatment of patients with acute ischemic stroke. Previous works have shown that collateral imaging, derived from CT angiography, MR perfusion, and MR angiography, aids in evaluating the collateral status. However, such methods are time-consuming and/or sub-optimal due to the nature of manual processing and heuristics. Recently, deep learning approaches have shown to be promising for generating collateral imaging. These, however, suffer from the computational complexity and cost.

**Methods:**

In this study, we propose a mobile, lightweight deep regression neural network for collateral imaging in acute ischemic stroke, leveraging dynamic susceptibility contrast MR perfusion (DSC-MRP). Built based upon lightweight convolution and Transformer architectures, the proposed model manages the balance between the model complexity and performance.

**Results:**

We evaluated the performance of the proposed model in generating the five-phase collateral maps, including arterial, capillary, early venous, late venous, and delayed phases, using DSC-MRP from 952 patients. In comparison with various deep learning models, the proposed method was superior to the competitors with similar complexity and was comparable to the competitors of high complexity.

**Conclusion:**

The results suggest that the proposed model is able to facilitate rapid and precise assessment of the collateral status of patients with acute ischemic stroke, leading to improved patient care and outcome.

## Introduction

In the diagnosis of patients with acute ischemic stroke (AIS), it has been shown that the collateral perfusion status plays a crucial role in cell viability and patient outcome [1]. There have been some research efforts to produce collateral imaging from various sources such as CT angiography [2, 3], dynamic susceptibility contrast-enhanced MR perfusion [4], and MR angiography [5]. Although these methods have shown to be effective in assessing the collateral status, they are, by and large, relying on manual operations and/or heuristic decisions/estimations by medical experts, which can be time-consuming and labor expensive. Furthermore, the assessment of the collateral status is subject to the level of expertise of clinicians, leading to discrepancies and instabilities in the diagnosis and treatment of patients [6]. Therefore, an alternative, accurate, and objective method that can assess the collateral status of patients is needed.

Recently, deep learning models have been successfully applied to a variety of applications in medical imaging [7–9]. Collateral imaging is not an exception. There exist some research efforts, based upon deep learning, which sought to produce collateral imaging without human intervention. For instance, [10] developed a 3D deep regression neural network (3D-DRNN) that can generate collateral imaging from dynamic susceptibility contrast MR perfusion (DSC-MRP). 3D-DRNN utilizes a notable encoder–decoder framework with 3D convolution kernels. [11] proposed 3D multi-task regression and ordinal regression deep neural network (3D-MROD-Net) to further improve the quality of collateral imaging by introducing ordinal regression and atrous spatial pyramid pooling (ASPP). The success of these previous methods is generally ascribable to the advances in the deep learning architecture, in particular with the increase in the model size and complexity, leading to increase in the cost for storage, computation, energy, and etc. [12, 13]. The same trend is often observed in other medical imaging applications [14] and other domains including computer vision [15], and natural language processing [16]. In order to alleviate such issues and to develop a compact and efficient model, tremendous research on a small, lightweight model is actively underway. For instance, SwinUNetR [17] and UNeXt [18] attained superior performance in medical image segmentation with a compact design of deep learning models.

**Fig. 1:**
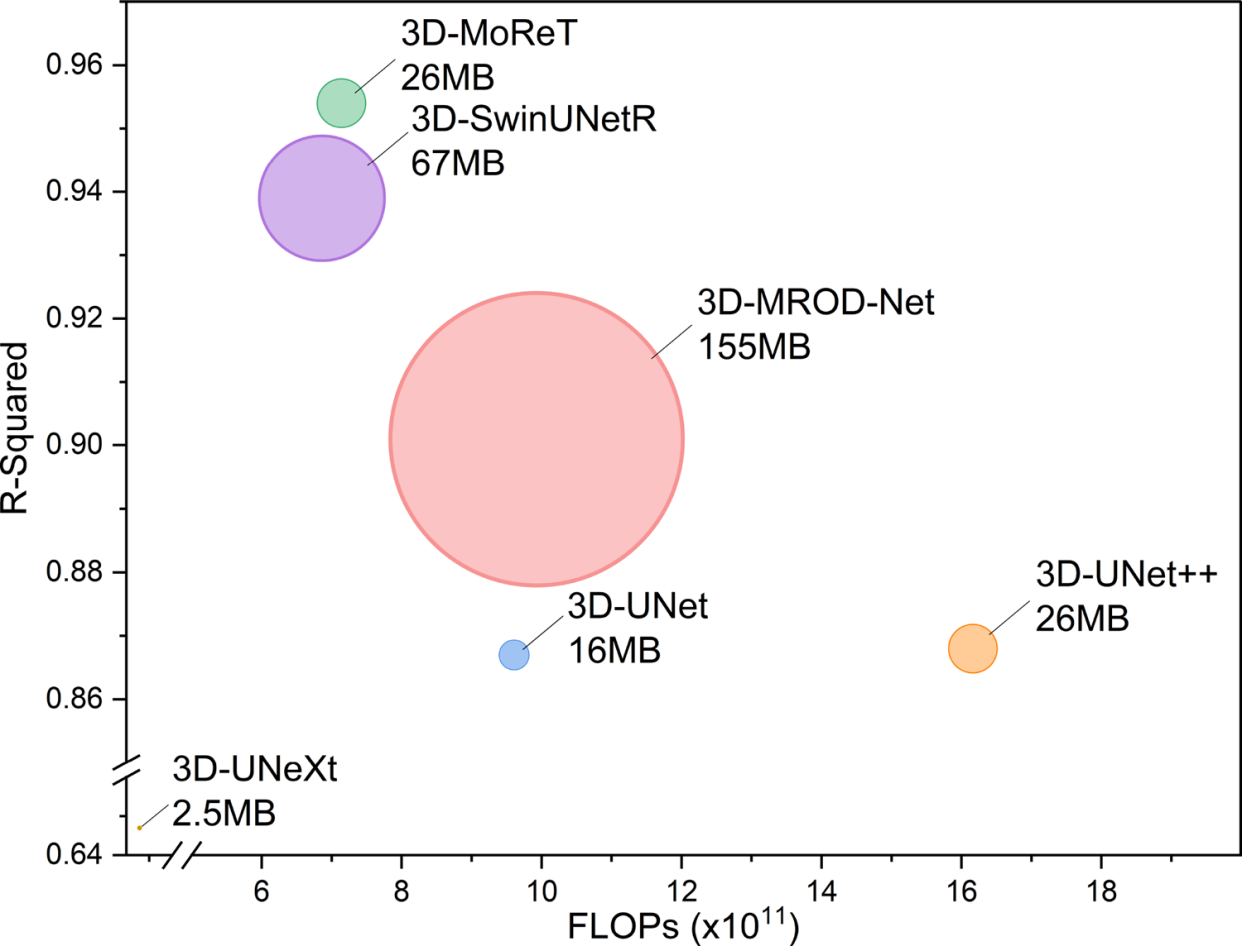
Computational complexity vs. performance. Floating-point operations per second (FLOPs), number of parameters, and R-Squared score are shown per deep learning model. The size of a circle corresponds to the number of parameters

Herein, we propose a lightweight but efficient model, called as 3D Mobile Regression vision Transformer (3D-MoReT), for collateral imaging from DSC-MRP. Following an encoder–decoder architecture, we build 3D-MoReT by devising and exploiting efficient convolution (Conv) and Transformer operations. For computational efficiency, the convolution operation takes advantages of depthwise (DW) and pointwise operations and the Transformer operation considers both local and global information/relationships. We employ DSC-MRP from 952 patients to systematically assess the efficiency and effectiveness of 3D-MoReT in comparison with a number of deep learning models that were previously built for collateral imaging, medical imaging, and computer vision. Figure 1 demonstrates that 3D-MoReT not only maintains low complexity in size and computation but also achieves the comparable performance to the state-of-the-art heavyweight deep learning model. The experimental results demonstrate that the proposed network achieves high performance in the generation of collateral maps as compared to other competing methods. Figure 3 demonstrates the workflow of the proposed method. The main contributions of the proposed approach can be summarized as follows:

- We propose a 3D lightweight model for the generation of the five-phase collateral maps from DSC-MRP images.
- We introduce a network architecture that is computational efficient and effective, and thus, it maintains the balance between the computational complexity and performance.
- We achieve superior performance on a dataset with 952 subjects as compared to 5 other competing models.

## Methods

### Datasets

**Table 1:** Data overview for training, validation, and test sets

	Train		Validation		Test	
	Stroke cases	Controls	Stroke cases	Controls	Stroke cases	Controls
# of subjects	400	46	150	19	143	194
Total	446		169		337

**Table 2:** MRI acquisition parameters

Parameters	SS EPI-DWI	SWI	DCE-MRA (TWIST)	DSC-MRP	T2-FLAIR
TR/TE (ms)	4800/71	28/20	2.62/0.95	1600/30	9000/95
TI (ms)			2500		
Turbofactor			21		
Flip angle		15$^{\circ }$	21$^{\circ }$	90$^{\circ }$	150$^{\circ }$
Bandwidth (Hz/pixel)	1672	120	780	1446	206
Slice thickness/gap (mm)	5/2	2/0	1.2/0	5/2	5/2
FOV (mm)	240 $\times$ 240	240 $\times$ 195	400 $\times$ 300	240 $\times$ 240	240 $\times$ 217
Matrix size (mm)	130 $\times$ 130	384 $\times$ 156	320 $\times$ 182	128 $\times$ 128	256 $\times$ 174
B-value (s/mm^2^)	0, 1000				
Measurements	1	1	30	60	1
GRAPPA	2	2	3 $\times$ 2	2	
Temporal resolution (s)			1.6	1.6	
Dynamic reconstruction mode			Forward share		
K-space sampling (center/periphery) (%)			15/20		

From November 2015 to March 2021, DSC-MRP images of 952 subjects were initially collected from two university medical centers (Table 1). Of these subjects, 693 are diagnosed with AIS (stroke cases), while the remaining 259 are diagnosed without AIS (controls). These subjects are divided into a training set, a validation set, and a test set. The training set, validation set, and test set include 446 subjects (400 strokes cases and 46 controls), 169 subjects (150 stroke cases and 19 controls), and 337 subjects (143 stroke cases and 194 controls), respectively. The primary purpose of the training set is to adjust the weights of deep learning models for collateral imaging. The validation set is used to determine the optimal weights, which are used for the evaluation on the test set.

**Fig. 2:**
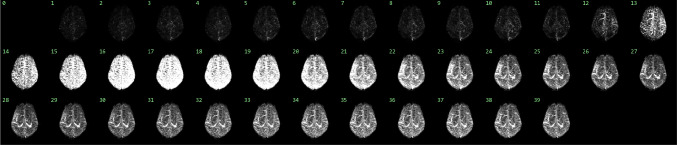
subtracted Maximum Intensity Projection (sMIP) image across the 40 time series to depict the dynamic changes in blood flow

**Fig. 3:**
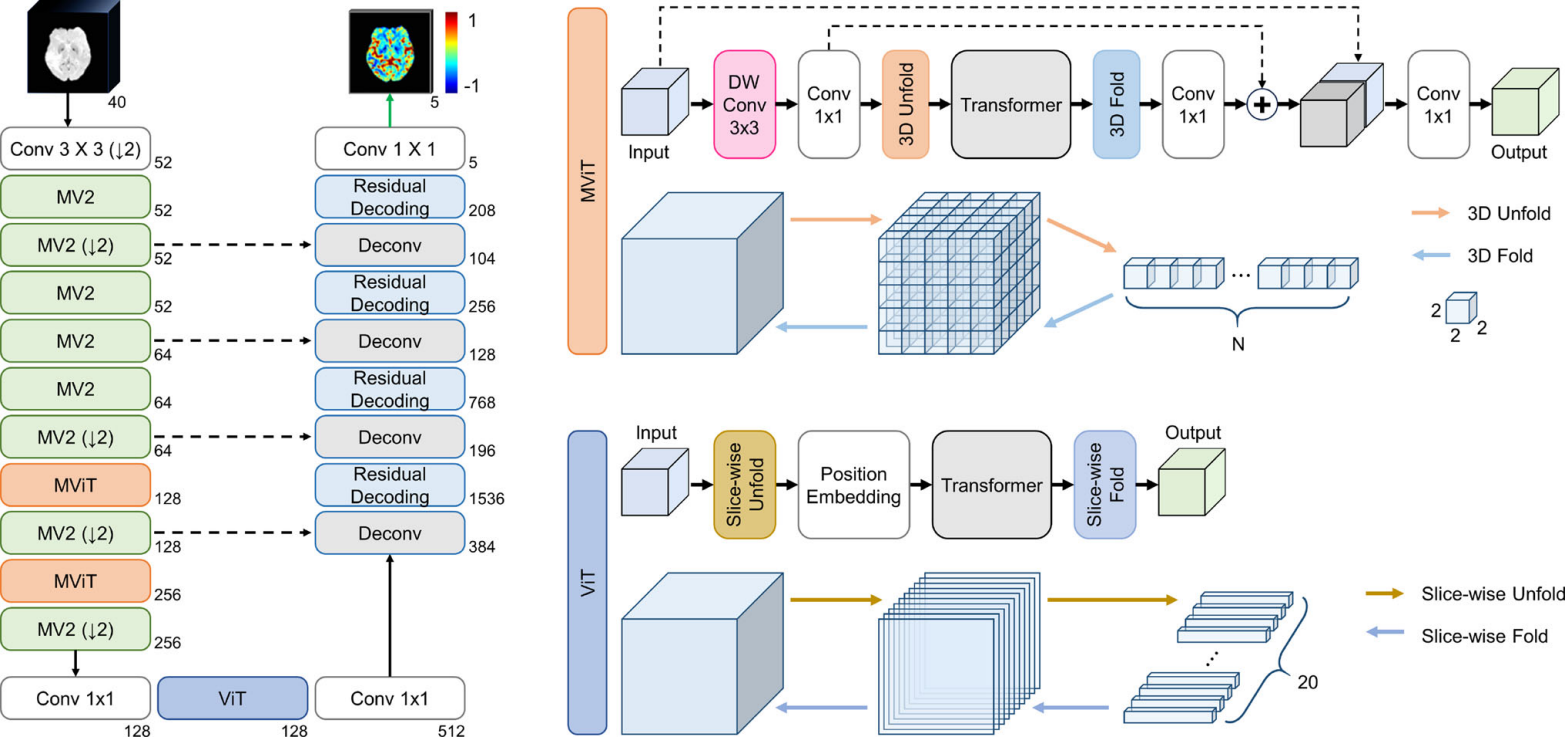
Overview of 3D-MoReT. 3D-MoReT is an encoder–decoder structure, which is built based upon convolution layers, MV2 blocks, MViT blocks, a ViT block, deconvolution layers, and residual layers

### MR imaging protocol

All subjects underwent MRI by 3T MAGNETOM Skyra MRI system (Siemens Healthcare) and Ingenia system (Philips Healthcare). Diffusion-weighted Imaging (DWI), Susceptibility-weighted MRI (SWI), Dynamic contrast-enhanced MR angiography (DCE-MRA) using TWIST, DSC-MRP, and T2-weighted fluid-attenuated inversion recovery (FLAIR) were acquired. The total MRI duration was 6 min 36 s using 7.5 ml of gadobutrol contrast. Detailed parameters are available in Table 2.

### Collateral imaging

An in-house MATLAB-based software is employed to semi-automatically generate collateral imaging from DSC-MRP. The software reads DSC-MRP DICOM images, extracts the brain area, produces maximum intensity projection (MIP) images by subtracting the first non-contrast phase image for each time point, which shows the progression of the bolus (contrast media) through each phase, chooses regions of interest (ROIs) for detailed examination on the middle cerebral artery (MCA) and the superior sagittal sinus (SSS), plots signal intensity–time curves using these ROIs to visualize the arterial and venous dynamic blood flow, and generates collateral maps with five phases. The five phases include (1) arterial phase (Art): from the arrival of contrast agent at the MCA to the arterial peak; (2) capillary phase (Cap): between the arterial peak and the venous peak in the SSS; (3) early venous phase (EVen): from the first half of the venous phase to the beginning of the venous plateau; (4) late venous phase (LVen): the second half of the venous phase; (5) delay phase (Del): after the venous phase. For each subject, the ground truth collateral imaging is generated by two expert neuro-radiologists (H.J.K and H.G.R.).

### Preprocessing

Each DSC-MRP is 4-dimensional (channel $\times$ depth $\times$ height $\times$ width), where the channel represents time series, the depth indicates the number of slices within each time series, and height and width denote the spatial size. The spatial size is 230 $\times$ 230. The number of channel and depth is 40 and 20, respectively. For computational efficiency, each DSC-MRP undergoes the following preprocessing steps.

(1) *Binary mask creation:* in order to exclude unnecessary areas, we create a binary mask by summing up the DSC-MRP images across all time points. This summed image is then normalized between 0 and 1 (min-max normalization). We then apply a threshold of 0.1 to the normalized images and refine the thresholded area with morphological operations such as closing, erosion, and dilation using a spherical kernel with a radius of 6.

(2) *Image normalization:* for improved contrast and accuracy, we normalize DSC-MRP image by 0-1 normalization within a brain mask and collateral maps within a range of $-$0.9 to 0.9 as outliers are clipped to either 0 or 1.

(3) *Filtering:* to further improve the signal-to-noise ratio, we apply median filtering with a kernel size of 5 to the collateral maps.

(4) *Cropping:* we center crop DSC-MRP images and collateral maps to a spatial size of 224 $\times$ 224.

Upon completion of the preprocessing steps, we obtain a 4-dimensional sub-volume of size 40 $\times$ 20 $\times$ 224 $\times$ 224 (channel $\times$ depth $\times$ height $\times$ width).

## Network architecture

Following the encoder–decoder architecture, we construct 3D-MoReT by utilizing MobileNet V2 (MV2) blocks [19], MobileViT blocks (MViT) [20], a ViT layer [21], residual layers, and skip connections. Figure 3 illustrates the overview of the architecture.

### Encoder

Motivated by [22], the encoder contains four groups of computing blocks, each of which is built based upon (1) a 3D convolution layer, (2) 3D MV2 blocks, (3) 3D MViT and 3D MV2 blocks, and (4) a ViT block and 3D convolution layers. The first group contains a 3D convolution layer with 1 x 3 x 3 kernels. The second group includes two sets of 2 3D MV2 blocks with stride 1 and a 3D MV2 block with stride 2. The third group consists of two sets of a 3D MViT block and a 3D MV2 block with stride 2. The last group has a ViT block, which is proceeded and followed by a 3D pointwise convolution layer. Combining these four groups of computing blocks, the encoder converts the input DSC-MRP of size 40 x 20 x 224 x 224 into the feature map of size 512 x 20 x 6 x 6.

The 3D MV2 block has a stack of pointwise–depthwise–pointwise 3D convolution layers. With a stride 2 in the depthwise convolution, we conduct the spatial down-sampling by a factor of 2. In the 3D MViT block, the input goes through 3D depthwise–pointwise convolution layers and $L_M$ 3D Transformer layers, followed by a pointwise convolution layer. $L_M$ is set to 2 and 4 for the first and second 3D MViT blocks, respectively. The input to the Transformer layers is added to its output, i.e., a residual layout. The resultant output is concatenated with the initial input to the 3D MViT block and undergoes another pointwise convolution layer to produce the final output of the 3D MViT block. Before the input tensor $x \in \mathbb {R}^{C \times D \times H \times W}$ is fed into the Transformer layers, we unfold *x* into *N* non-overlapping volumes of size $p \times p \times p$, producing $x^{U} \in \mathbb {R}^{C \times N \times P}$ where $P=p^3$ and $N=\frac{D \times H \times W}{P}$. Then, the output of the Transformer layers is folded to recover the original shape of the tensor, producing $x^{F} \in \mathbb {R}^{C \times D \times H \times W}$. We set *p* to 2. The Transformer layers conduct multi-head self-attention [23] with 4 heads.

**Table 3:** Results of the prediction of the five-phase collateral maps. Numbers represent average and standard deviation over all patients

Model	Metric	Art	Cap	EVen	LVen	Del
**3D-MoReT**	R-Squared	**0.926 ± 0.102**	**0.974 ± 0.053**	**0.974 ± 0.045**	**0.955 ± 0.060**	**0.940 ± 0.067**
3D-MROD-Net		0.838 ± 0.248	0.942 ± 0.122	0.941 ± 0.105	0.904 ± 0.134	0.879 ± 0.137
3D-UNet		0.758 ± 0.247	0.917 ± 0.112	0.922 ± 0.098	0.883 ± 0.122	0.856 ± 0.127
3D-UNet++		0.769 ± 0.240	0.920 ± 0.101	0.925 ± 0.086	0.878 ± 0.122	0.849 ± 0.132
3D-SwinUNetR		0.894 ± 0.100	0.964 ± 0.050	0.964 ± 0.043	0.944 ± 0.057	0.926 ± 0.067
3D-UNeXt		0.607 ± 0.102	0.693 ± 0.057	0.695 ± 0.052	0.638 ± 0.067	0.603 ± 0.069
**3D-MoReT**	MAE	0.066 ± 0.030	0.048 ± 0.020	**0**.**051** ± **0**.**022**	**0**.**069** ± **0**.**024**	0.076 ± 0.028
3D-MROD-Net		**0**.**059** ± **0**.**032**	**0**.**043** ± **0**.**021**	0.052 ± 0.023	0.069 ± 0.027	**0**.**075** ± **0**.**030**
3D-UNet		0.078 ± 0.032	0.055 ± 0.020	0.058 ± 0.022	0.075 ± 0.026	0.082 ± 0.029
3D-UNet++		0.075 ± 0.031	0.054 ± 0.019	0.057 ± 0.021	0.076 ± 0.026	0.084 ± 0.029
3D-SwinUNetR		0.090 ± 0.030	0.062 ± 0.020	0.077 ± 0.023	0.088 ± 0.023	0.098 ± 0.024
3D-UNeXt		0.192 ± 0.031	0.166 ± 0.018	0.185 ± 0.011	0.203 ± 0.011	0.209 ± 0.011
**3D-MoReT**	SSIM	0.913 ± 0.101	0.966 ± 0.056	**0.970 ± 0.044**	**0.948 ± 0.060**	**0.934 ± 0.066**
3D-MROD-Net		**0.923 ± 0.109**	**0.969 ± 0.065**	0.969 ± 0.056	0.947 ± 0.078	0.933 ± 0.081
3D-UNet		0.880 ± 0.112	0.957 ± 0.054	0.960 ± 0.052	0.938 ± 0.069	0.923 ± 0.072
3D-UNet++		0.885 ± 0.105	0.958 ± 0.051	0.962 ± 0.046	0.935 ± 0.068	0.921 ± 0.072
3D-SwinUNetR		0.841 ± 0.106	0.951 ± 0.054	0.941 ± 0.047	0.924 ± 0.061	0.903 ± 0.067
3D-UNeXt		0.541 ± 0.087	0.617 ± 0.060	0.560 ± 0.058	0.487 ± 0.066	0.477 ± 0.062
**3D-MoReT**	TM	0.947 ± 0.046	**0**.**978** ± **0**.**031**	0.978 ± 0.024	0.963 ± 0.032	0.955 ± 0.037
3D-MROD-Net		**0**.**954** ± **0**.**048**	0.921 ± 0.031	**0**.**979** ± **0**.**028**	**0**.**964** ± **0**.**033**	**0**.**956** ± **0**.**039**
3D-UNet		0.931 ± 0.046	0.973 ± 0.027	0.972 ± 0.026	0.955 ± 0.034	0.948 ± 0.038
3D-UNet++		0.929 ± 0.048	0.973 ± 0.027	0.972 ± 0.023	0.950 ± 0.034	0.945 ± 0.039
3D-SwinUNetR		0.909 ± 0.046	0.969 ± 0.024	0.959 ± 0.024	0.946 ± 0.033	0.936 ± 0.033
3D-UNeXt		0.811 ± 0.032	0.828± 0.024	0.743 ± 0.058	0.680 ± 0.071	0.704 ± 0.059

Numbers in bold represent the highest values obtained per phase using each evaluation metric

**Fig. 4:**
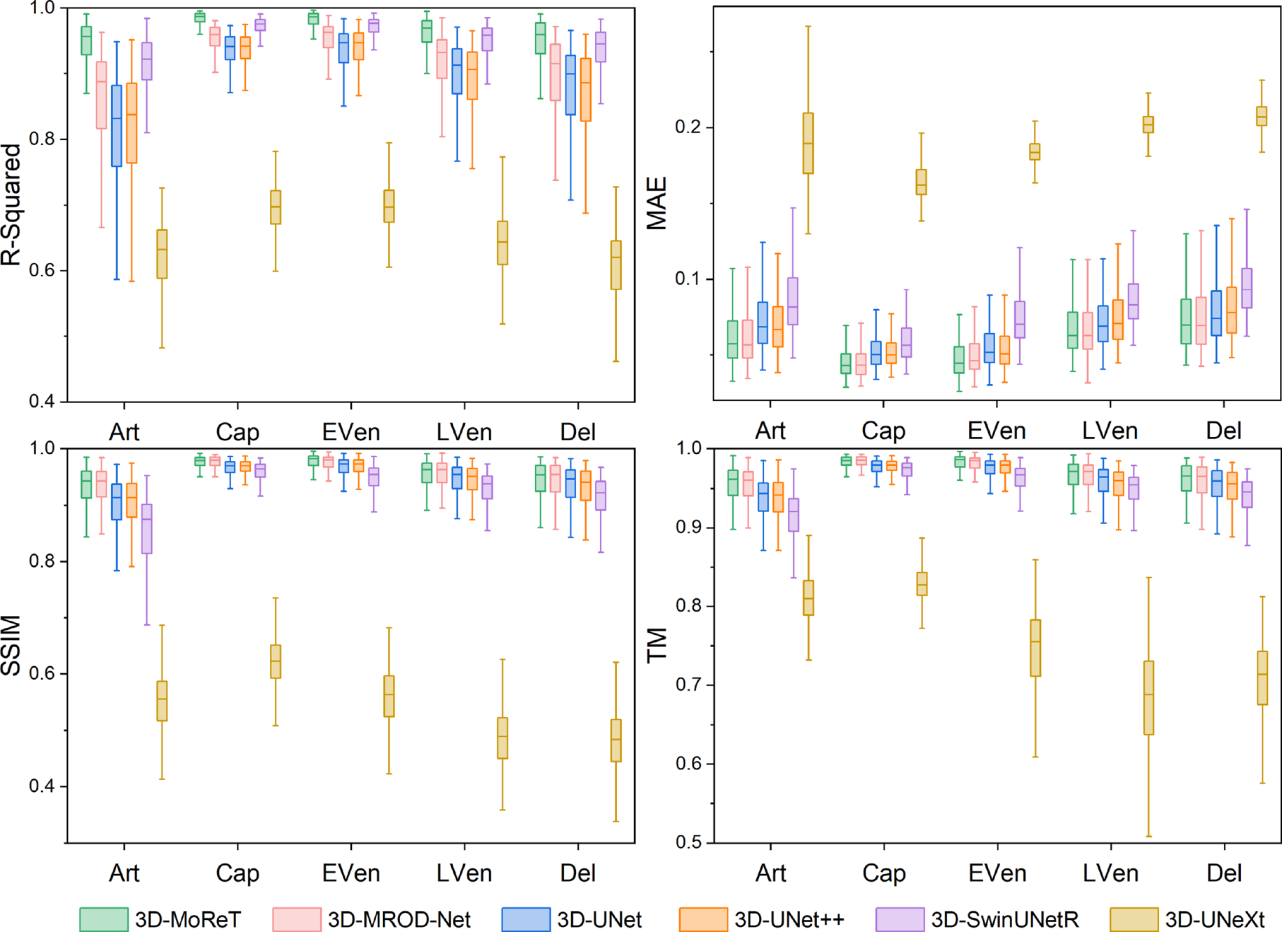
Boxplots of R-Squared, MAE, SSIM, and TM between the ground truth and the predicted phase maps on the test set

The ViT block comprises of slice-wise unfolding, a position embedding [24], and $L_V$ Transformer layers. The slice-wise unfolding (or patch embedding) transforms the input tensor $x \in \mathbb {R}^{C \times D \times H \times W}$ into $x^U \in \mathbb {R}^{C \times N \times P}$ where $N=D$ and $P=H \times W$. The position embedding adds the positional information per patch. The number of heads is set to 4 in the Transformer layers. The output of the Transformer layers is slice-wise folded, generating the output tensor $x^F \in \mathbb {R}^{C \times D \times H \times W}$.

#### Decoder

The decoder consists of five sets of a deconvolution (Deconv) layer and a residual layer. The deconvolution layer employs a 3 $\times$ 3 $\times$ 3 transposed convolution, which doubles the spatial dimension of the feature maps. The output of the deconvolution layer is concatenated with the output of the corresponding encoder layer through a skip connection and is fed into the residual layer. This residual layer includes a sequence of 3 $\times$ 1 $\times$ 1, 3 $\times$ 3 $\times$ 3, and 3 $\times$ 1 $\times$ 1 convolutions with a short connection linking its input to its output. Finally, it utilizes a 1 $\times$ 1 $\times$ 1 convolution layer with five channels, followed by a Tanh activation function, to predict the five-phase collateral maps. The Tanh activation function produces the real-valued output within the [$-$1, 1] range for each phase, i.e., conducting a regression task.

### Loss function

To optimize 3D-MoReT, we adopt BerHu loss [25], which can be defined as follows:

1 $$\begin{aligned} L(y, f(x)) = {\left\{ \begin{array}{ll} |y - f(x)| &{} \text {if } |y - f(x)| \le c \\ \frac{(y - f(x))^2 + c^2}{2c} &{} \text {otherwise} \end{array}\right. } \end{aligned}$$

where $y$ is the ground truth, $f(x)$ is the predicted value, and $c$ is the threshold, which is set to 0.2. L1 and L2 loss functions are popular choices for regression problems; however, these are known to have issues with non-differentiable behavior and vulnerability to outliers. BerHu loss has shown to increase the robustness against outliers, which can occur in patient data variability, leading to enhanced generalization capabilities of the model.

### Network training and selection strategy

We optimize 3D-MoReT with Adam optimizer and the initial learning rate of 0.001, which decreased by a factor of 0.75 if there is no improvement over 3 epochs. The size of mini-batch is set to 4. Only horizontal flipping is performed for data augmentation due to the left-right symmetry in human brain structures. We train 3D-MoReT for 300 epochs on the training dataset and the optimal model, minimizing the validation loss, is chosen to be tested on the test dataset.

All the training and inference are performed on a PC with Intel$^{\circledR }$ Xeon$^{\circledR }$ Silver 4210R processors, 64 GB of RAM, and 1 NVIDIA RTX A6000 48 GB Graphics Processing Unit (GPU).

### Evaluation metrics

To evaluate the performance of 3D-MoReT, several evaluation metrics are utilized, including the squared of the correlation coefficient (R-Squared), mean absolute error (MAE), Tanimoto measure (TM), and structural similarity index measure (SSIM). We compute these metrics for each of the five-phase collateral maps per subject. Then, the average and standard deviation over all subjects are calculated.

Suppose that $\hat{y} = \{\hat{y}_i\}_{i=1}^{n}$ and $y = \{y_i\}_{i=1}^{n}$ is the predicted and ground truth collateral maps , where *i* and *n* denote each voxel and the total number of voxels, respectively. R-Squared, ranging from 0 to 1, quantifies how well the ground truth is explained by the prediction, which is defined as follows:

2 $$\begin{aligned} R-Squared = 1 - \frac{\sum _{i=1}^{n} (y_i - \hat{y}_i))^2}{\sum _{i=1}^{n} (y_i - \bar{y})^2} \end{aligned}$$

where $\bar{y}$ is the mean of *y*.

MAE measures the average of the absolute differences between $\hat{y}$ and *y*, which is given by:

3 $$\begin{aligned} MAE = \frac{1}{n} \sum _{i=1}^{n} |y_i - \hat{y}_i| \end{aligned}$$

TM utilizes Euclidean distance between $\hat{y}$ and *y*. TM ranges from 0 to 1, and the smaller distance is, the higher TM is. TM is computed as follows:

4 $$\begin{aligned} TM = \frac{\sum _{i=1}^{n} (y_i \cdot \hat{y_i})}{\sum _{i=1}^{n} (y_i^2) + \sum _{i=1}^{n} (\hat{y_i}^2) - \sum _{i=1}^{n} (y_i \cdot \hat{y}_i)} \end{aligned}$$

SSIM quantifies the similarity between $\hat{y}$ and *y* in regard with the inter-dependencies among pixels/voxels, which is formulated as:

5 $$\begin{aligned} SSIM = \frac{(2 \mu _{\hat{y}} \mu _y + C_1)(2 \sigma _{\hat{y}y} + C_2)}{(\mu _{\hat{y}}^2 + \mu _y^2 + C_1)(\sigma _{\hat{y}}^2 + \sigma _y^2 + C_2)} \end{aligned}$$

where $\mu _{\hat{y}}$ and $\mu _y$ represents the mean of $\hat{y}$ and *y*, respectively. $\sigma _{\hat{y}}^2$ and $\sigma _y^2$ are the variance of $\hat{y}$ and *y*, respectively. $\sigma _{\hat{y}y}$ is the covariance between $\hat{y}$ and *y*. $C_1$ and $C_2$ are small constants added to avoid division by zero. SSIM ranges from $-$1 to 1. The higher SSIM, the greater similarity between $\hat{y}$ and *y* is.

**Table 4:** Results of the prediction of the five-phase collateral maps per subject group. Numbers represent average and standard deviation over all patients

Diagnosis class	Metric	Art	Cap	EVen	LVen	Del
Stroke	R-Squared	0.9334 ± 0.0773	0.9774 ± 0.0530	0.9779 ± 0.0339	0.9612 ± 0.0288	0.9424 ± 0.0594
	MAE	0.0630 ± 0.0256	0.0459 ± 0.0182	0.0487 ± 0.0191	0.0665 ± 0.0171	0.0757 ± 0.0262
	SSIM	0.9214 ± 0.0772	0.9698 ± 0.0522	0.9734 ± 0.0335	0.9546 ± 0.0289	0.9364 ± 0.0586
	TM	0.9508 ± 0.0399	0.9811 ± 0.0222	0.9807 ± 0.0193	0.9661 ± 0.0195	0.9557 ± 0.0345
Control	R-Squared	0.9255 ± 0.1019	0.9744 ± 0.0532	0.9743 ± 0.0445	0.9550 ± 0.0602	0.9396 ± 0.0665
	MAE	0.0657 ± 0.0301	0.0481 ± 0.0200	0.0508 ± 0.0224	0.0686 ± 0.0243	0.0764 ± 0.0279
	SSIM	0.9128 ± 0.1010	0.9661 ± 0.0554	0.9696 ± 0.0440	0.9484 ± 0.0598	0.9337 ± 0.0659
	TM	0.9475 ± 0.0457	0.9783 ± 0.0314	0.9784 ± 0.0240	0.9628 ± 0.0323	0.9545 ± 0.0365

**Table 5:** Results of the prediction of the five-phase collateral maps per medical center. Numbers represent average and standard deviation over all patients

Medical center	Metric	Art	Cap	EVen	LVen	Del
$\#$ 1	R-Squared	0.9387 ± 0.0729	0.9784 ± 0.0327	0.9767 ± 0.0330	0.9551 ± 0.0594	0.9425 ± 0.0600
	MAE	0.0608 ± 0.0225	0.0452 ± 0.0171	0.0497 ± 0.0191	0.0664 ± 0.0271	0.0739 ± 0.0261
	SSIM	0.9260 ± 0.0713	0.9714 ± 0.0336	0.9718 ± 0.0333	0.9501 ± 0.0590	0.9374 ± 0.0584
	TM	0.9529 ± 0.0316	0.9812 ± 0.0184	0.9795 ± 0.0216	0.9646 ± 0.0361	0.9575 ± 0.0340
$\#$ 2	R-Squared	0.9245 ± 0.1038	0.9741 ± 0.0545	0.9741 ± 0.0452	0.9550 ± 0.0603	0.9393 ± 0.0670
	MAE	0.0661 ± 0.0306	0.0483 ± 0.0202	0.0509 ± 0.0227	0.0688 ± 0.0241	0.0766 ± 0.0281
	SSIM	0.9118 ± 0.1029	0.9657 ± 0.0568	0.9694 ± 0.0448	0.9482 ± 0.0598	0.9334 ± 0.0664
	TM	0.9470 ± 0.0467	0.9780 ± 0.0322	0.9783 ± 0.0241	0.9627 ± 0.0319	0.9543 ± 0.0367

### Comparative experiments

To evaluate the performance of 3D-MoReT, we employ a number of deep learning models including three CNN models and two Transformer models. The three CNN models are 3D-UNet [26], 3D-UNet++, and 3D-MROD-Net [11]. The two Transformer models are 3D-SwinUNetR [17] and 3D-UNeXt [18].

3D-UNet [26] is a 3D version of UNet [27] that is built based upon an encoder–decoder architecture. This follows the same architecture of UNet but replaces all 2D convolutions with 3D convolutions. 3D-UNet++ is a 3D version of UNet++ [28], which extends UNet by redesigning skip connections. Instead of the direction connection between the corresponding layers in the encoder and decoder, UNet++ introduces a series of nested dense convolutions among the encoder and decoder. It also employs deep supervision for enhanced performance. 3D-MROD-Net is a deep learning model that is built specifically for collateral imaging. Similar to UNet and UNet++, 3D-MROD-Net follows the encoder–decoder architecture and adopts EfficientNet [29] as an encoder and ASPP in between the encoder and decoder to utilize the multi-scale semantic information. It approaches collateral map prediction as a regression and an ordinal regression. 3D-SwinUNetR is a 3D version of SwinUNetR [17], which employs Swin transformer [30] as an encoder to extract features at five different resolutions by utilizing shifted windows for computing self-attention. And each resolution level is then connected to an FCNN-based decoder via skip connections. It can enhance the model’s capacity for detailed feature extraction and processing in three-dimensional space. And 3D-UNeXt is a 3D version of UNeXt [18], which is designed with an initial convolution stage and a subsequent MLP stage. It uses a tokenized MLP block for effective processing of convolutional features and focuses on local dependencies by shifting input channels. This method reduces parameters and complexity while improving regression. Additionally, the network integrates skip connections between the encoder and decoder.

**Fig. 5:**
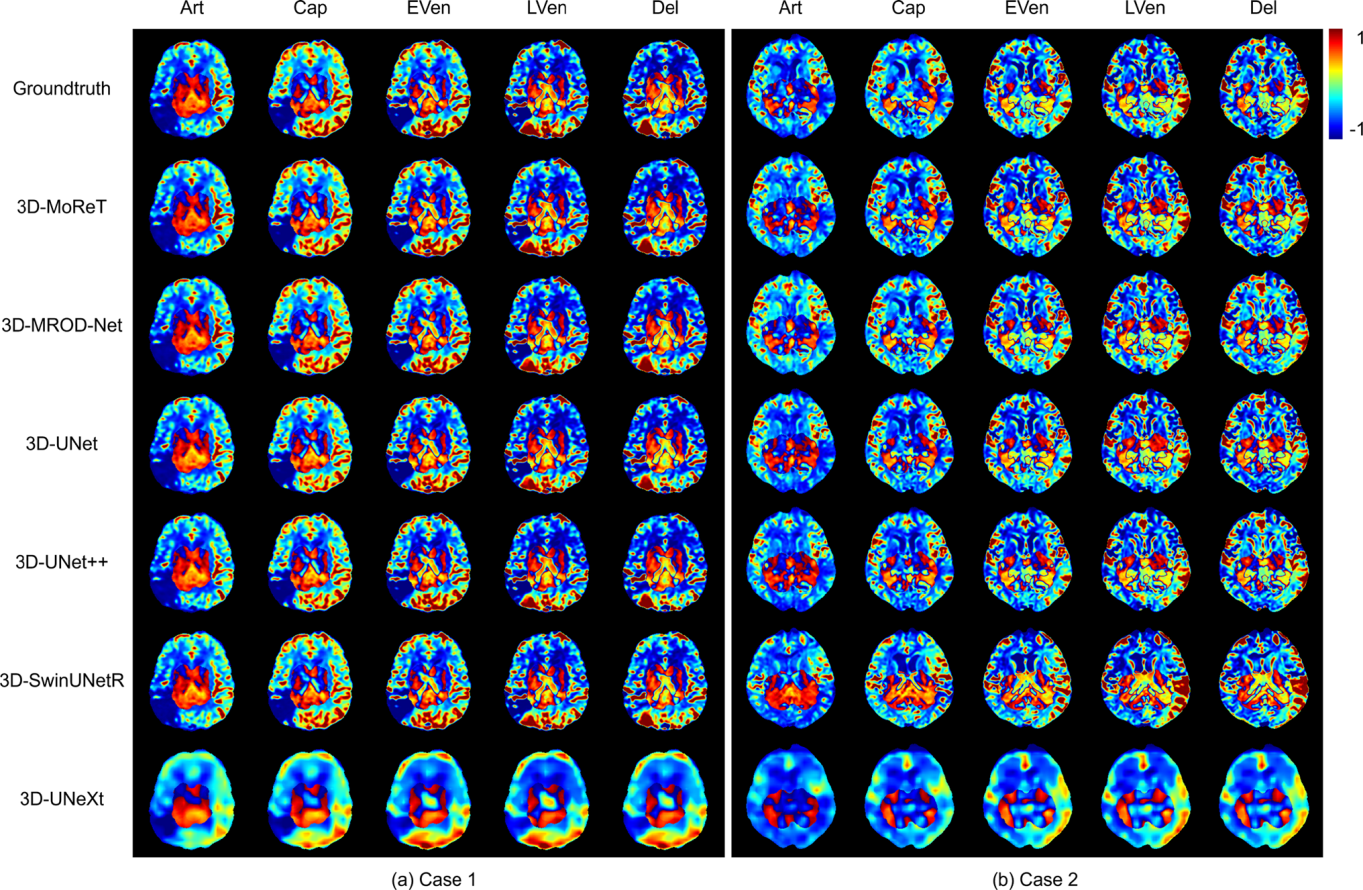
Prediction of five-phase collateral maps on stroke cases

We make modifications to the comparative models to adapt them for the collateral map generation, using a Tanh activation function at the last layer.

## Results and discussions

Table 3 and Fig. 4 demonstrate the results of the generation of the five-phase collateral maps by the proposed 3D-MoReT and other competitors. Overall, 3D-MoReT was able to generate the five-phase collateral maps with high accuracy; for instance, on average, $\ge$0.926 R-Squared, $\le$0.048 MAE, $\ge$0.913 SSIM, and $\ge$0.947 TM were achieved across the five phases by 3D-MoReT. Moreover, in a head-to-head comparison against other competitors, the superior ability of 3D-MoReT in generating the five-phase collateral maps was confirmed. For example, 3D-MoReT obtained the best performance in all five phases by R-Squared, 2 phases (EVen and LVen) by MAE, 3 phases (EVen, LVen, and Del) by SSIM, and 1 phase (Cap) by TM. For other phases, 3D-MoReT was consistently the second-best model regardless of the evaluation metrics.

To evaluate the potential biases in the performance, we further investigated the prediction results by 3D-MoReT in two ways: (1) Comparison across two diagnostic classes: stroke cases vs. control cases and (2) Comparison between two medical centers. Table 4 demonstrates the performance of the five-phase collateral maps generated by 3D-MoReT per diagnostic class. The difference between the two diagnostic classes was minimal for all evaluation metrics, suggesting that 3D-MoReT is able to handle both stroke and control cases in a reliable and accurate manner. Table 5 shows the comparison between two university medical centers. The performance of 3D-MoReT was slightly better in Medical Center $\#$1 using all evaluation metrics across the five phases; however, the difference was negligible. These observations demonstrate the robustness of 3D-MoReT under diverse settings, holding potential for clinical usage.

**Fig. 6:**
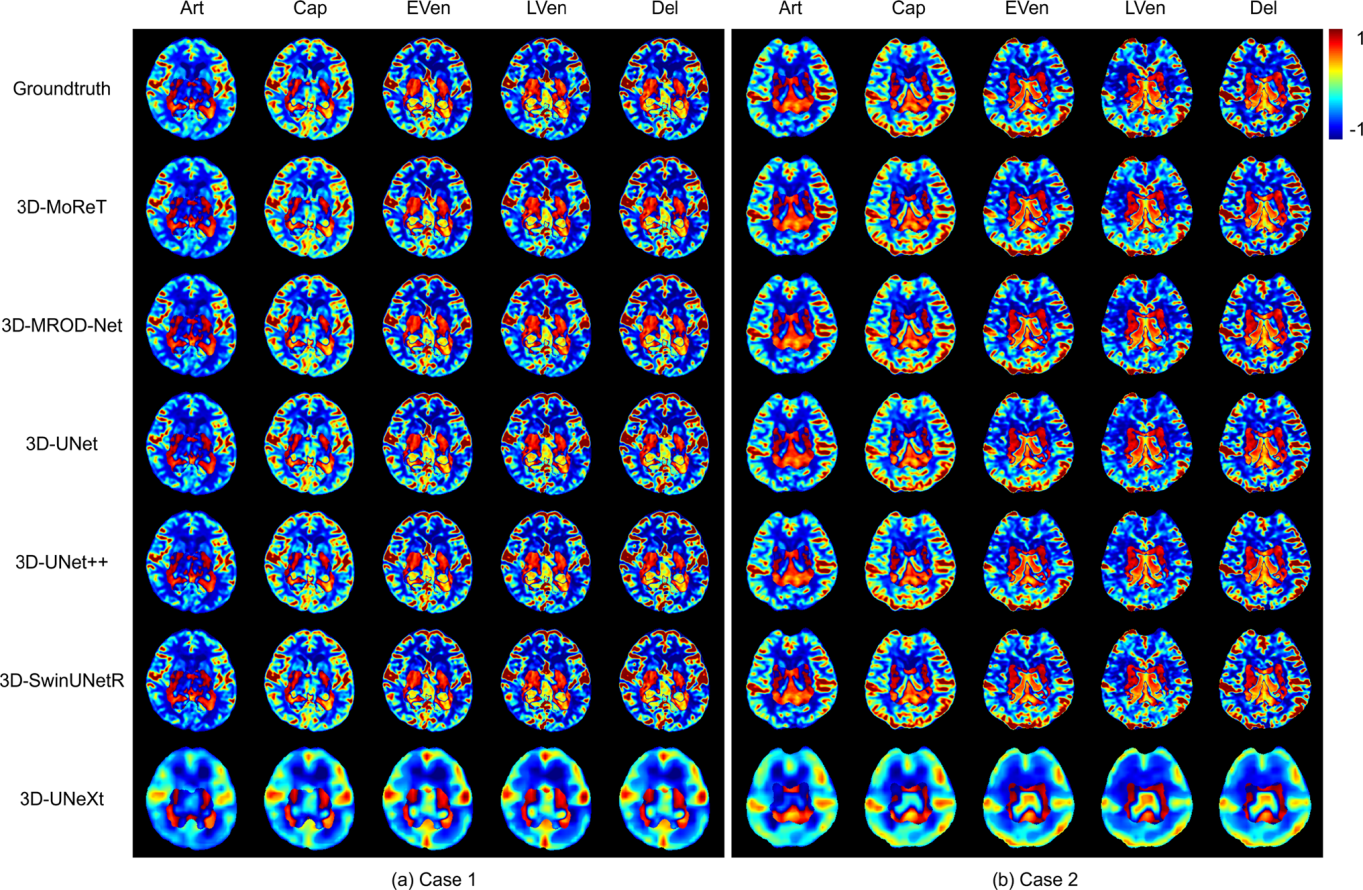
Prediction of five-phase collateral maps on control cases

To qualitatively assess the prediction results, we visualize and analyze the predicted collateral maps by 3D-MoReT and other competitors. Figure 5 shows the two exemplary stroke cases, and Fig. 6 depicts the two exemplary controls cases. We observed that the collateral maps generated by 3D-MoReT were highly correlated with the ground truth collateral maps across the five phases and patients. The predicted collateral maps clearly show the infarct lesion and collateral perfusion status in stroke cases. There was no prominent difference between the stroke cases and control cases with respect to the quality of the collateral maps. These suggest that 3D-MoReT is able to generate realistic collateral maps that can visually assess the collateral status. The predicted collateral maps by the second-best model, 3D-MROD-Net, were similar to those by 3D-MoReT. However, the overall quality of the predicted collateral maps by other competitors was disproportionate across the five phases.

Furthermore, we investigated the computational complexity of 3D-MoReT and other competitors using the number of parameters and size, floating-point operations per second (FLOPs), and inference time. The results are available in Table 6. 3D-MoReT was the third smallest model with respect to the number of parameters and size as well as was the third most efficient model in terms of FLOPs and inference time. 3D-UNeXt and 3D-UNet were the two smallest models and 3D-UNeXt and 3D-SwinUNetR were the two most efficient models. However, the performance of these competing models was substantially lower than that of 3D-MoReT. We also note that 3D-MROD-Net, obtained the second-best performance, was computational inefficient, requiring $\times$6 parameters and size, 40% increase in FLOPs, and $\times$2 inference time as compared to 3D-MoReT. These results suggest that 3D-MoReT achieves both the accurate performance and the computational efficiency.

In both quantitative and qualitative analyses of the prediction results, the performance of 3D-MoReT surpassed other competing models. 3D-MoReT was also computationally efficient compared to others. The advantages of 3D-MoReT can be ascribable to its efficient and effective design. It is equipped with a mobile convolution (MV2) block, a mobile ViT (MViT) block, a ViT block, a convolutional layer, a deconvolution layer, and a residual layer. In particular, MV2 blocks enable 3D-MoReT to extract low-level features with low-cost, MViT blocks allow it to learn and exploit the relationship among 3-dimensional sub-volumes, and ViT facilitates the interactions among slices. As combined together, these can enhance the overall understanding of the 4-dimensional image context, leading to accurate predictions.

This study has several limitations. First, 3D-MoReT was trained and tested using a private dataset obtained from two medical centers due to the lack of public datasets. An extended study needs to be followed to further validate our findings. Second, we used DSC-MRP to generate collateral maps. There exist other imaging modalities that can be used to generate collateral maps. Third, the clinical implications of the predicted collateral maps have not yet been investigated. Though previous studies have demonstrated the usefulness of collateral maps [31, 32], the predicted or AI-generated collateral maps have not been investigated with respect to the diagnostic accuracy and reliability in clinics. The future study will include the clinical evaluation of 3D-MoReT. Last, there is an imbalance in the performance, notably in the prediction of the artery phase. We will explore proper strategies to achieve improved results.

**Table 6:** Computational complexity of 3D-MoReT and other competing models

Model	# of Params ($\times 10^{3}$)	Model size (MB)	FLOPs ($\times 10^{11}$)	Inference time (s)
3D-MoReT	26.736	25.512	7.140	3.560
3D-MROD-NET	162.543	155.013	9.929	7.575
3D-UNet	16.375	15.617	9.606	5.619
3D-UNet++	27.500	26.226	16.163	9.739
3D-SwinUNetR	62.254	66.551	6.860	1.310
3D-UNeXt	2.667	2.543	2.873	0.008

## Conclusions

In this study, we present an approach of a lightweight deep neural network, designated as 3D-MoReT, for efficient and effective collateral imaging in acute ischemic stroke. Exploiting the recent advances in deep learning, in particular transformers, 3D-MoReT was able to generate the five-phase collateral maps from DSC-MRP in an accurate and reliable fashion, meanwhile maintaining the low computational complexity and cost. For the superior performance and efficiency, 3D-MoReT could facilitate improved diagnosis of acute ischemic stroke in clinics. Moreover, 3D-MoReT has potential for broader applications in other areas of biomedical imaging. The capability of the model to learn complex patterns and features of cerebral blood flow could enhance the diagnostic accuracy in various kinds of vascular diseases such as coronary artery disease, peripheral artery disease, and aortic aneurysm. The efficient and effective design of the model could facilitate the development of AI systems embedded in medical devices. A direct application to different imaging types, e.g., CT, ultrasound, and etc., may require specific modifications and adaptations of the model architecture, particularly in preprocessing steps. In the follow-up study, we will further develop and optimize the model for both performance and computational efficiency and conduct extended validations to comprehensively evaluate its clinical impact. We will also explore the application of 3D-MoReT in other types of medical imaging and disease to fully assess its potential and adaptability.

## References

[1] Menon BK, Smith EE, Modi J, Patel SK, Bhatia R, Watson TWJ, Hill MD, Demchuk AM, Goyal M (2011) Regional leptomeningeal score on ct angiography predicts clinical and imaging outcomes in patients with acute anterior circulation occlusions. Am J Neuroradiol 32:1640–1645. 10.3174/ajnr.A2564 21799045 10.3174/ajnr.A2564 PMC7965388

[2] Broocks G, Kemmling A, Meyer L, Nawabi J, Schön G, Fiehler J, Kniep H, Hanning U (2019) Computed tomography angiography collateral profile is directly linked to early edema progression rate in acute ischemic stroke. Stroke 50:3424–3430. 10.1161/STROKEAHA.119.027062 31665994 10.1161/STROKEAHA.119.027062

[3] Sallustio F, Motta C, Pizzuto S, Diomedi M, Giordano A, D’Agostino V, Samà D, Mangiafico S, Saia V, Legramante J, Konda D, Pampana E, Floris R, Stanzione P, Gandini R, Koch G (2017) Ct angiography-based collateral flow and time to reperfusion are strong predictors of outcome in endovascular treatment of patients with stroke. J Neurointerventional Surg 9:940–943. 10.1136/neurintsurg-2016-012628 10.1136/neurintsurg-2016-012628 27663559

[4] Nael K, Doshi A, Leacy RD, Puig J, Castellanos M, Bederson J, Naidich TP, Mocco J, Wintermark M (2018) Mr perfusion to determine the status of collaterals in patients with acute ischemic stroke: A look beyond time maps. Am J Neuroradiol 39:219–225. 10.3174/ajnr.A5454 29217747 10.3174/ajnr.A5454 PMC7410580

[5] Maguida G, Shuaib A (2023) Collateral circulation in ischemic stroke: an updated review. J Stroke. 10.5853/jos.2022.02936 36907186 10.5853/jos.2022.02936 PMC10250877

[6] Tetteh G, Navarro F, Meier R, Kaesmacher J, Paetzold JC, Kirschke JS, Zimmer C, Wiest R, Menze BH (2023) A deep learning approach to predict collateral flow in stroke patients using radiomic features from perfusion images. Front Neurol. 10.3389/fneur.2023.1039693 36895903 10.3389/fneur.2023.1039693 PMC9990868

[7] Anaya-Isaza A, Mera-Jiménez L, Zequera-Diaz M (2021) An overview of deep learning in medical imaging. Inf Med Unlocked 26:100723. 10.1016/j.imu.2021.100723

[8] Aggarwal R, Sounderajah V, Martin G, Ting DSW, Karthikesalingam A, King D, Ashrafian H, Darzi A (2021) Diagnostic accuracy of deep learning in medical imaging: a systematic review and meta-analysis. NPJ Digit Med 4:65. 10.1038/s41746-021-00438-z 33828217 10.1038/s41746-021-00438-z PMC8027892

[9] Chen X, Wang X, Zhang K, Fung K-M, Thai TC, Moore K, Mannel RS, Liu H, Zheng B, Qiu Y (2022) Recent advances and clinical applications of deep learning in medical image analysis. Med Image Anal 79:102444. 10.1016/j.media.2022.102444 35472844 10.1016/j.media.2022.102444 PMC9156578

[10] Nhat TMN, Jeong KH, Gee RH, Yoon-Sik C, Tae KJ (2020) Deep regression neural networks for collateral imaging from dynamic susceptibility contrast-enhanced magnetic resonance perfusion in acute ischemic stroke. Int J Comput Assist Radiol Surg 15:151–162. 10.1007/s11548-019-02060-7 31482272 10.1007/s11548-019-02060-7

[11] Long LH, Gee RH, Jeong KH, Tae KJ (2022) A 3d multi-task regression and ordinal regression deep neural network for collateral imaging from dynamic susceptibility contrast-enhanced mr perfusion in acute ischemic stroke. Comput Methods Progr Biomed. 10.1016/j.cmpb.2022.107071 10.1016/j.cmpb.2022.107071 35994873

[12] Hu X, Chu L, Pei J, Liu W, Bian J (2021) Model Complexity of Deep Learning: A Survey. 10.48550/arXiv.2103.05127. https://api.semanticscholar.org/CorpusID:232168493

[13] Horowitz M (2014) 1.1 Computing’s energy problem (and what we can do about it). 10.1109/ISSCC.2014.6757323. https://api.semanticscholar.org/CorpusID:232168493

[14] Celard P, Iglesias E, Fdez JM, Romero R, Vieira S, Borrajo M (2022) A survey on deep learning applied to medical images: from simple artificial neural networks to generative models. Neural Comput Appl 35:1–33. 10.1007/s00521-022-07953-4 36373133 10.1007/s00521-022-07953-4 PMC9638354

[15] Chai J, Zeng H, Li A, Ngai EWT (2021) Deep learning in computer vision: a critical review of emerging techniques and application scenarios. Mach Learn Appl 6:100134. 10.1016/j.mlwa.2021.100134

[16] Desislavov R, Martínez-Plumed F, Hernández-Orallo J (2023) Trends in ai inference energy consumption: Beyond the performance-vs-parameter laws of deep learning. Sustain Comput: Inf Syst **38**. 10.1016/j.suscom.2023.100857

[17] Hatamizadeh A, Nath V, Tang Y, Yang D, Roth H, Xu D (2022) Swin UNETR: Swin transformers for semantic segmentation of brain tumors in MRI images . 10.48550/arXiv.2201.01266. https://api.semanticscholar.org/CorpusID:245668780

[18] Valanarasu JMJ, Patel VM (2022) UNeXt: MLP-based Rapid Medical Image Segmentation Network . 10.48550/arXiv.2203.04967. https://api.semanticscholar.org/CorpusID:247362702

[19] Sandler M, Howard AG, Zhu M, Zhmoginov A, Chen L-C (2018) Mobilenetv2: Inverted residuals and linear bottlenecks. 2018 IEEE/CVF Conference on Computer Vision and Pattern Recognition, 4510–4520 10.48550/arXiv.1801.04381

[20] Mehta S, Rastegari M (2022) MobileViT: Light-weight, General-purpose, and Mobile-friendly Vision Transformer. 10.48550/arXiv.2110.02178. https://openreview.net/forum?id=vh-0sUt8HlG

[21] Dosovitskiy A, Beyer L, Kolesnikov A, Weissenborn D, Zhai X, Unterthiner T, Dehghani M, Minderer M, Heigold G, Gelly S, Uszkoreit J, Houlsby N (2020) An image is worth 16x16 words: Transformers for image recognition at scale. CoRR arXiv:2010.11929. 10.48550/arXiv.2010.11929

[22] Wadekar SN, Chaurasia A (2023) MobileViTv3: Mobile-Friendly Vision Transformer with Simple and Effective Fusion of Local, Global and Input Features. 10.48550/arXiv.2209.15159. https://openreview.net/forum?id=wtr-9AKxCI5

[23] Cordonnier J, Loukas A, Jaggi M (2020) Multi-head attention: Collaborate instead of concatenate. CoRR arXiv:2006.16362. 10.48550/arXiv.2006.16362

[24] Su J, Lu Y, Pan S, Wen B, Liu Y (2021) Roformer: Enhanced transformer with rotary position embedding. CoRR arXiv:2104.09864. 10.48550/arXiv.2104.09864

[25] Zwald L, Lambert-Lacroix S (2012) The berhu penalty and the grouped effect. arXiv: Statistics Theory. 10.48550/arXiv.1207.6868

[26] Çiçek, Abdulkadir A, Lienkamp SS, Brox T, Ronneberger O (2016) 3d u-net: Learning dense volumetric segmentation from sparse annotation. International Conference on Medical Image Computing and Computer-Assisted Intervention. 10.48550/arXiv.1606.06650

[27] Ronneberger O, Fischer P, Brox T (2015) U-net: Convolutional networks for biomedical image segmentation. Medical image computing and computer-assisted intervention-MICCAI 2015: 18th international conference. 10.48550/arXiv.1505.04597

[28] Zhou Z, Siddiquee MMR, Tajbakhsh N, Liang J (2018) Unet++: A nested u-net architecture for medical image segmentation. Deep Learning in Medical Image Analysis and Multimodal Learning for Clinical Decision Support : 4th International Workshop, DLMIA 2018, and 8th International Workshop, ML-CDS 2018, held in conjunction with MICCAI 2018, Granada, Spain, S. 11045, 3–11 10.48550/arXiv.1807.10165 10.1007/978-3-030-00889-5_1 PMC7329239 32613207

[29] Tan M, Le QV (2019) Efficientnet: Rethinking model scaling for convolutional neural networks, 6105–6114. 10.48550/arXiv.1905.11946

[30] Liu Z, Lin Y, Cao Y, Hu H, Wei Y, Zhang Z, Lin S, Guo B (2021) Swin transformer: Hierarchical vision transformer using shifted windows. CoRR arXiv:2103.14030. 10.48550/arXiv.2103.14030

[31] Jeon YS, Kim HJ, Roh HG, Lee T-J, Park JJ, Lee SB, Lee HJ, Kwak JT, Lee JS, Ki HJ (2024) Impact of collateral circulation on futile endovascular thrombectomy in acute anterior circulation ischemic stroke. J Korean Neurosurg Soc. 10.3340/jkns.2023.0139 10.3340/jkns.2023.0139 PMC10788550 37536707

[32] Yi JS, Ki HJ, Jeon YS, Park JJ, Lee T-J, Kwak JT, Lee SB, Lee HJ, Kim IS, Kim JH, Lee JS, Roh HG, Kim HJ (2024) The collateral map: prediction of lesion growth and penumbra after acute anterior circulation ischemic stroke. Eur Radiol. 10.1007/s00330-023-10084-6 37646808 10.1007/s00330-023-10084-6 PMC10873223

